# Percutenous Catheter Ablation of the Accessory Pathway in a Patient with Wolff-Parkinson-White Syndrome Associated with Familial Atrial Fibrillation

**Published:** 2008-04-01

**Authors:** Serkan Cay, Serkan Topaloglu, Dursun Aras

**Affiliations:** Department of Cardiology, Yuksek Ihtisas Heart-Education and Research Hospital, Ankara, Turkey

**Keywords:** Ablation, cardiac arrest, familial atrial fibrillation, WPW syndrome

## Abstract

Percutenous catheter ablation of the accessory pathway in Wolff-Parkinson-White syndrome is a highly successful mode of therapy. Sudden cardiac arrest survivors associated with WPW syndrome should undergo radiofrequency catheter ablation. WPW syndrome associated with familial atrial fibrillation is a very rare condition. Herein, we describe a case who presented with sudden cardiac arrest secondary to WPW syndrome and familial atrial fibrillation and treated via radiofrequency catheter ablation.

Wolff-Parkinson-White (WPW) is an abnormality of the conduction tissue in which an accessory pathway conducts atrial depolarizations together with normal conduction pathway at the same time. Therefore, a short PR interval, a delta wave, and a widened QRS complex are seen on the surface electrocardiography. If a documented tachycardia is present, the pathology is called as WPW syndrome. Percutaneous catheter ablation of the accessory pathway has been used to treat this type of tachycardias with high success rates [1]. Herein, we describe a case with WPW syndrome who presented with ventricular fibrillation secondary to familial atrial fibrillation whose accessory pathway was ablated.

## Case report

**Figure 1:**
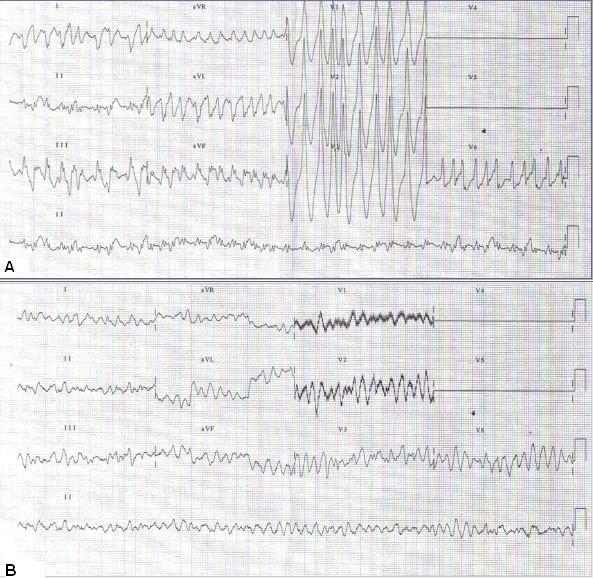
Twelve-lead ECG showed a wide QRS complex tachycardia resembling ventricular tachycardia (**A**) that degenerated to ventricular fibrillation (**B**).

**Figure 2:**
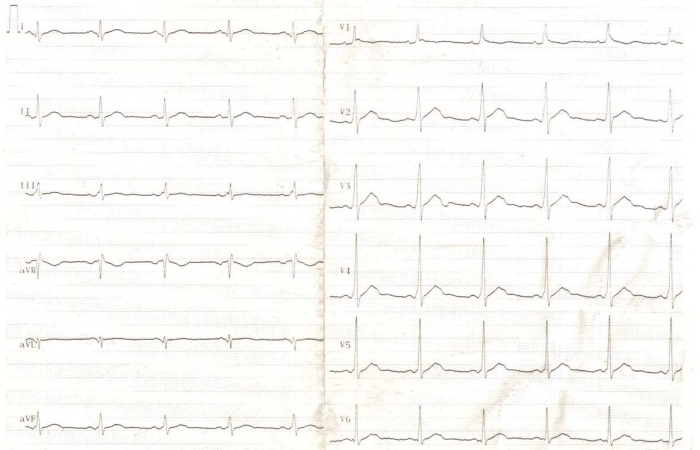
Twelve-lead ECG on admission showed WPW pattern with negative delta waves in leads I and aVL, and positive delta waves in leads II, III, aVF, and precordial leads.

**Figure 3:**
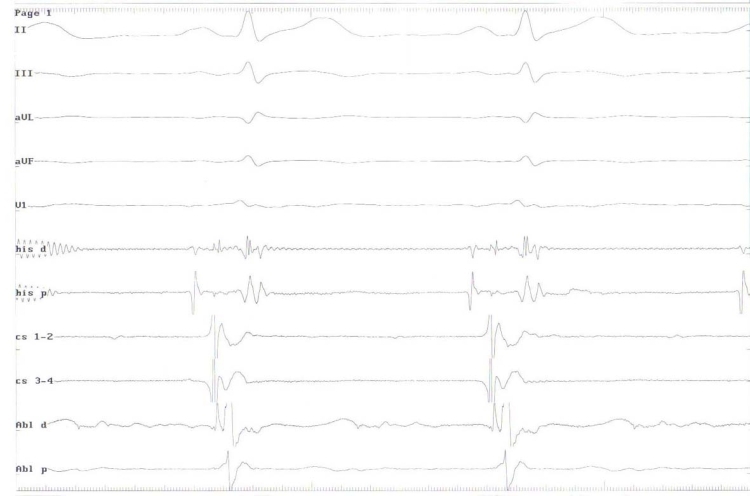
Intracardiac electrograms showed a very short HV interval at both distal coronary sinus and ablation catheter sites consistent with a left lateral accessory pathway. Abl, Ablation catheter electrograms; cs, Coronary sinus electrograms; his, Bundle of His electrograms; H, His; V, Ventricle.

**Figure 4:**
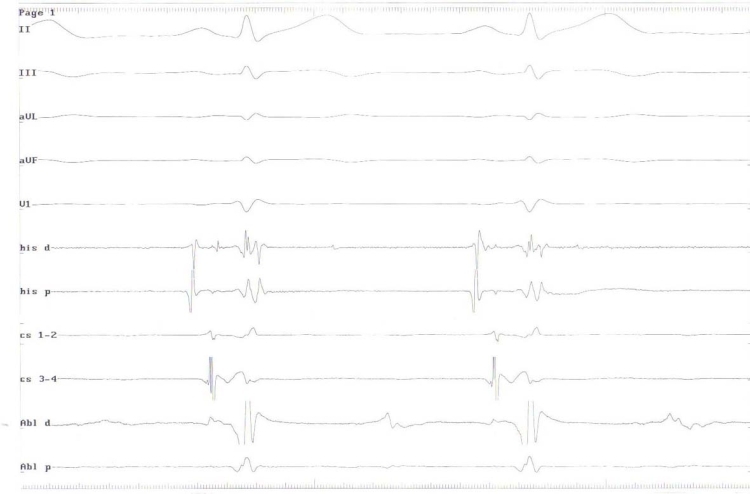
Intracardiac electrograms showed a lengthened HV interval at both distal coronary sinus and ablation catheter sites that mean successful ablation of the accessory pathway. Abl, Ablation catheter electrograms; cs, Coronary sinus electrograms; his, Bundle of His electrograms; H, His; V, Ventricle.

Previously asymptomatic 36-year-old male had been admitted to another clinic due to severe palpitation and syncope while watching television. After arrival to the emergency department, sudden cardiac arrest had developed because of ventricular fibrillation. The first ECG showed a wide QRS tachycardia and the second one revealed ventricular fibrillation (Figure 1). He was defibrillated twice and sinus rhythm with hemodynamic stability  achieved. Then, the patient was referred to our clinic for further evaluation. Family members with lone atrial fibrillation were recognized from the patient's history. In addition, his uncle had died suddenly during sleep at the age of 32 years. Electrocardiograms of the family members revealed normal sinus rhythm without WPW pattern. Laboratory tests and echocardiography were unremarkable. The electrocardiogram on admission showed WPW pattern with prominent delta waves, short PR intervals, and widened QRS complexes especially in leads V2, V3, and V4. Negative delta waves in leads I and aVL, and positive delta waves in both V1 and V2 with a "pseudo-right bundle branch block" QRS complex appearance with positive QRS complex (Figure 2). Two 6F steerable multielectrode quadripolar recording catheters positioned at bundle of His area, and in the coronary sinus with the proximal electrode pair at the coronary sinus ostium (LivewireTM, St. Jude Medical, USA) via femoral vein were used for mapping. Programmed atrial and ventricular stimulation and incremental atrial and ventricular pacing are performed. A left lateral atrioventricular accessory pathway was detected (Figure 3). The refractory period of the accessory pathway was 230 msec. Radio frequency ablation was performed using a radiofrequency generator (ATAKR® II, Medtronic, USA) via a radiofrequency catheter (RF Marinr® MR, Medtronic, USA). The His bundle, the coronary sinus and the ablation site electrograms were recorded at a paper rate of 100 mm/sec. Catheter ablation for the left-sided atrioventricular accessory pathway was performed via the arterial approach retrogradely. The optimal ablation site was determined and radiofrequency energy was delivered for 60 seconds at a temperature of 60°C. After the first delivery of radiofrequency energy, the delta wave disappeared and HV intervals in the coronary sinus electrograms lengthened (Figure 4). Stimulation protocols were made to induce reciprocating tachycardia; however, no tachycardia was induced. The patient has been asymptomatic for 6 months and no delta wave has appeared.

## Discussion

Patients with WPW syndrome may have several mechanisms of tachyarrhythmia. AF occurs frequently in patients with WPW syndrome. WPW syndrome itself is not related to atrial disease, which may provide an electrophysiologic substrate for AF. The occurrence of clinical AF in patients with WPW syndrome usually correlates with the presence of concomitant AV reciprocating tachycardia. Transition of AV reciprocating tachycardia to AF has been observed in electrophysiologic studies, with AF usually initiated by atrial extrasystoles [2]. In addition, AF does not recur after successful ablation of the accessory pathway. Intrinsic atrial abnormalities, short atrial refractory periods, rapid AV reciprocating tachycardia rates, alterations in excitation-contraction feedback, and a participatory role of the accessory pathway may be the factors related to atrial fibrillation. Anterograde conduction via the AV node during AF is generally limited to a rate of 180 to 200 beats/min; however, accessory pathways with short refractory periods might conduct more rapid atrial depolarizations like atrial fibrillation causing rapid ventricular rate and finally ventricular fibrillation and sudden cardiac arrest as in our patient. Ventricular fibrillation as the first manifestation of the syndrome may be seen although patients with WPW are usually asymptomatic [3]. The most recent study [4] reports a sudden cardiac death risk of 0.02%/patient/year. Recent studies have provided evidence of a genetic contribution to AF. Mutations in potassium-channel genes have been associated with familial AF [5]. A recent survey in a large cohort of lone AF demonstrated that 39% of individuals had a positive family history [6].

In conclusion, patients with WPW pattern is under risk of adverse cardiac events including sudden cardiac death. This risk is higher in WPW patients with familial AF. Therefore, percutenous catheter ablation of WPW patients, especially prone to atrial fibrillation, even in asymptomatic stages should be performed when the diagnosis has been made. In addition, surveillance of the family members for familial AF should be conducted.
